# A novel time-lapse imaging method for studying developing bacterial biofilms

**DOI:** 10.1038/s41598-022-24431-y

**Published:** 2022-12-07

**Authors:** Momir Futo, Tin Široki, Sara Koska, Nina Čorak, Anja Tušar, Mirjana Domazet-Lošo, Tomislav Domazet-Lošo

**Affiliations:** 1 Faculty of Electrical Engineering and Computing, University of Zagreb, Unska 3, 10000 Zagreb, Croatia; 2 Laboratory of Evolutionary Genetics, Division of Molecular Biology, Ruđer Bošković Institute, Bijenička Cesta 54, 10000 Zagreb, Croatia; 3 School of Medicine, Catholic University of Croatia, Ilica 242, 10000 Zagreb, Croatia

**Keywords:** Time-lapse imaging, Biofilms, Evolutionary developmental biology

## Abstract

In nature, bacteria prevailingly reside in the form of biofilms. These elaborately organized surface-bound assemblages of bacterial cells show numerous features of multicellular organization. We recently showed that biofilm growth is a true developmental process, which resembles developmental processes in multicellular eukaryotes. To study the biofilm growth in a fashion of eukaryotic ontogeny, it is essential to define dynamics and critical transitional phases of this process. The first step in this endeavor is to record the gross morphological changes of biofilm ontogeny under standardized conditions. This visual information is instrumental in guiding the sampling strategy for the later omics analyses of biofilm ontogeny. However, none of the currently available visualizations methods is specifically tailored for recording gross morphology across the whole biofilm development. To address this void, here we present an affordable Arduino-based approach for time-lapse visualization of complete biofilm ontogeny using bright field stereomicroscopy with episcopic illumination. The major challenge in recording biofilm development on the air–solid interphase is water condensation, which compromises filming directly through the lid of a Petri dish. To overcome these trade-offs, we developed an Arduino microcontroller setup which synchronizes a robotic arm, responsible for opening and closing the Petri dish lid, with the activity of a stereomicroscope-mounted camera and lighting conditions. We placed this setup into a microbiological incubator that maintains temperature and humidity during the biofilm growth. As a proof-of-principle, we recorded biofilm development of five *Bacillus subtilis* strains that show different morphological and developmental dynamics.

## Introduction

Bacterial biofilms are the most common life-form on Earth^1,2^. They are highly organized surface-associated communities of bacterial cells embedded in a self-derived extracellular matrix^3,4^. From the origin of life, bacterial biofilms are continuously present in the most diverse habitats where they show stunning ecological adaptations^5–7^. Due to their ecological, medical^8,9^ and commercial importance^10,11^, bacterial biofilms have been studied from many angles using state-of-the-art omics approaches^12–14^. As an example, we recently showed that biofilm growth represents a true developmental process that contains discrete developmental phases comparable to those of developing eukaryotic embryos^14^.

To achieve these findings, we transferred experimental and analytical approaches regularly used in developmental biology of multicellular eukaryotes to the analysis of *Bacillus subtilis* biofilm ontogeny^14–17^. Traditionally, the study of eukaryotic development starts with a careful description of gross morphological changes along the ontogeny; from fertilization until the formation of an adult organism^18,19^. To ensure reproducibility and allow comparisons across studies, the developmental process of a eukaryotic organism is usually divided into stages that are temporally and morphologically defined under standardized laboratory conditions^18,19^. However, these developmental stages not only help researchers to navigate along the developmental process, but also reflect underlying molecular processes that are organized in a discrete fashion^14,16,20–22^. It is, therefore, important that the gross morphological changes of biofilm ontogeny are recorded and evaluated before the biofilm sampling for various omics analyses is performed.

**Figure 1:**
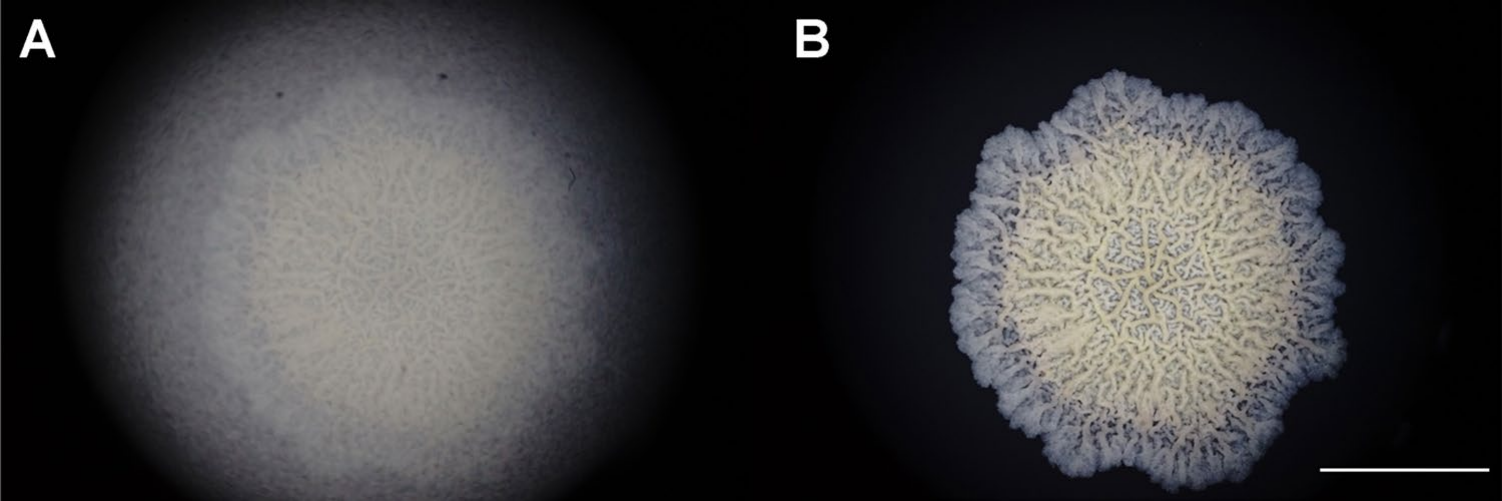
Water condensation on the Petri dish lid obstructs visual clarity during time-lapse visualization of *Bacillus subtilis* biofilm development. We photographed a 3 days old *Bacillus subtilis* NCIB 3610 biofilm grown in a Petri dish on MSgg agar using a Zeiss Stemi C-2000 stereomicroscope with episcopic (reflected) illumination (0.8 × magnification). The scale bar indicates 1 cm. The temporal dynamics of water condensation on a Petri dish lid is shown in Supplementary Video 1. (**A**) Photograph taken through the lid of a Petri dish obstructed by water condensation, (**B**) photograph of the same biofilm taken without the Petri dish lid.

To optimize the sampling strategy for downstream transcriptomic and proteomic analysis, we previously made a time-lapse video of complete *B. subtilis* development on a solid-air interface^14^. However, during these experiments we faced substantial methodological challenges related to the obstruction of visual clarity during the recording of time-lapse videos due to water condensation on Petri dish lids. In our experience, this is a prominent problem especially in the situation where low-magnification images are captured using bright field stereomicroscopy with episcopic (reflected) illumination (Fig. 1, Supplementary Video 1). The same condensation-induced photo blurriness was noticed previously in a comparable effort to record bacterial colony growth by time-lapse visualization^23^ and in attempts to photograph plant tissues cultured in Petri dishes^24^.

To address this issue, an earlier study suggested placement of a tick polymer disc with low thermal conductivity on the lid of a Petri dish that contains cultured plant tissues^24^. Although this approach reduced the condensation on the inner surface of the lid, it concomitantly increased condensation on the Petri dish side walls, which is an undesirable situation because it leads to excessive water dripping directly on the culturing media^24^. Another shortcoming of this solution is that adding a tick polymer disc in the optical path inevitably changes optical clarity, especially in the low-magnification setup with episcopic illumination that we used. Similarly, another study placed at the bottom and at the top of a Petri dish transparent glass heaters to avoid water condensation^25^. This approach was useful for recording transmission fluorescent signals during biofilm development, but it required a specialized and expensive fluorescence microscope for large object fields and a custom-built environmental chamber to maintain a constant temperature during growth^25^. In addition, glass heaters further reduce the light transmission and obstruct the optical path, especially if reflected illumination is applied.

An obvious solution to the lid-condensation problem would be to entirely remove the lid during the filming process. This approach also provides the least obstructed optical path if the bright field stereomicroscopy with episcopic illumination is applied. However, it also compromises the experiment by increasing desiccation and contamination of the agar medium in a Petri dish. In our experience, this leads to the reduction of usable recording time to at most three days. To address these tradeoffs, in our previous work we applied a brute-force approach by manually opening, filming and subsequently closing the Petri dish lid during the experiment^14^. However, this solution is rather impractical, or even prohibitive, as it required engagement of substantial workforce over the weeks-long filming sessions of biofilm development, which required shooting in 15-min intervals. Another drawback associated with this solution is that frequent opening and closing of the incubator door leads to temperature and humidity fluctuations, which in turn must be carefully controlled. In addition, the same procedure increases the probability of airborne contaminants in the incubator space.

To address these issues, we here present an Arduino microcontroller-based solution for time-lapse visualization of developing biofilms at solid-air interfaces using bright field stereomicroscopy with episcopic illumination. As a proof-of-concept, we recorded time-lapse videos of developing biofilms in five *B. subtilis* strains that show distinct biofilm morphologies and developmental dynamics.

## Results and discussion

### Technical aspects of the method

In a closed incubator environment, the standard growth temperature for mesophilic bacteria, which cover the majority of common environmental bacteria and human pathogens, ranges between 20 and 45 °C^26^. This relatively high incubator temperature causes an extensive water evaporation from agarose-based growth media, which often directly affects the growth of bacterial colonies. During standard bacterial incubations this excessive evaporation is prevented by a Petri dish lid. However, slight temperature differences between the lid and the inner Petri dish space often lead to moisture condensation on the inner Petri dish lid surface^24^. In the majority of microbiological experiments this phenomenon is of no concern, but in biofilm macrocolony photography and time-lapse video production, the vapor condensation on the transparent lid causes considerable difficulties in taking clear biofilm photographs (Fig. 1, Supplementary Video 1). This is also clearly visible in some time-lapse videos of a previous methodological paper that deals with bacterial macrocolony photography^23^.

**Figure 2:**
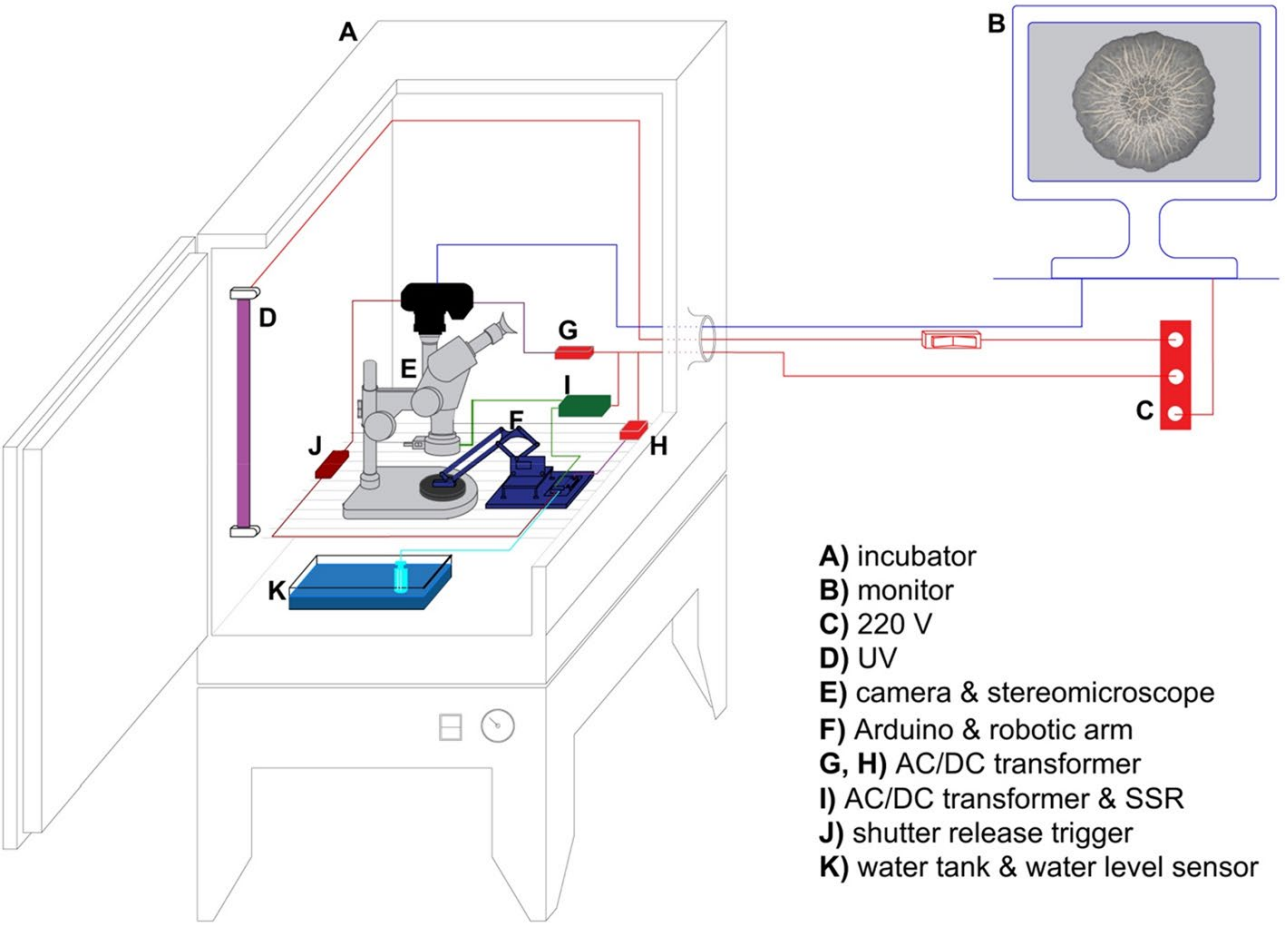
The Arduino-controlled biofilm visualization apparatus. (**A**) microbiological incubator, (**B**) an external computer monitor attached to the camera, (**C**) 220 V AC outlet, (**D**) UV lamp, (**E**) mirrorless SONY alpha 7 II camera coupled with a 144 LED light-ring mounted on a Zeiss Stemi C-2000 stereo microscope using a T2 adapter, (**F**) Arduino microcontroller with an acrylic robotic arm, (**G,H**) AC/DC transformers, (**I**) AC/DC transformer and a solid state relay module (Omron), (**J**) digital timer and remote shutter MC-36B (Neewer), (**K**) water-filled container with a water level detection sensor module.

**Figure 3:**
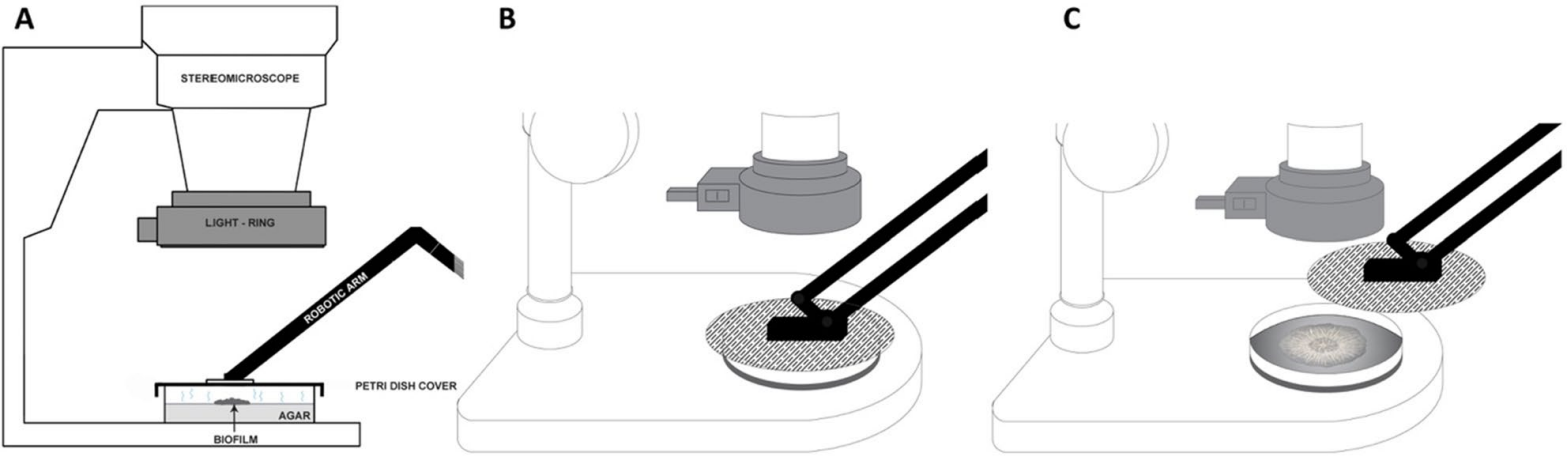
The principle of the robotic arm actions. (**A**) Sideview of the closed agar plate with a developing biofilm, (**B**) the closed Petri dish with a developing biofilm between shootings, (**C**) the robotic arm opens the Petri dish before the camera shooting will take place. Supplementary Video 2 shows the apparatus for automatic time-lapse recording and the robotic arm movements.

To overcome these obstacles, we constructed an Arduino-controlled biofilm visualization setup (Fig. 2). The core of this setup, which consists of a camera mounted on top of a stereomicroscope (Fig. 2E) and an Arduino microcontroller with robotic arm (Fig. 2F), is placed inside a microbiological incubator (Fig. 2A). The basic logic behind this apparatus is to automatize the Petri dish lid opening, synchronize this step with the automated camera shots of a growing biofilm, and perform these actions under tightly controlled temperature, humidity and lightning conditions. The main functional part in our setup is a small, acrylic robotic arm controlled by an Arduino microcontroller (Fig. 2F). The sole purpose of this robotic arm, which is firmly attached to the Petri dish lid, is to open and close the Petri dish in short, preprogramed photo-intervals that allow visually unobstructed shooting of a biofilm that grows on an agar plate (Fig. 3).

**Figure 4:**
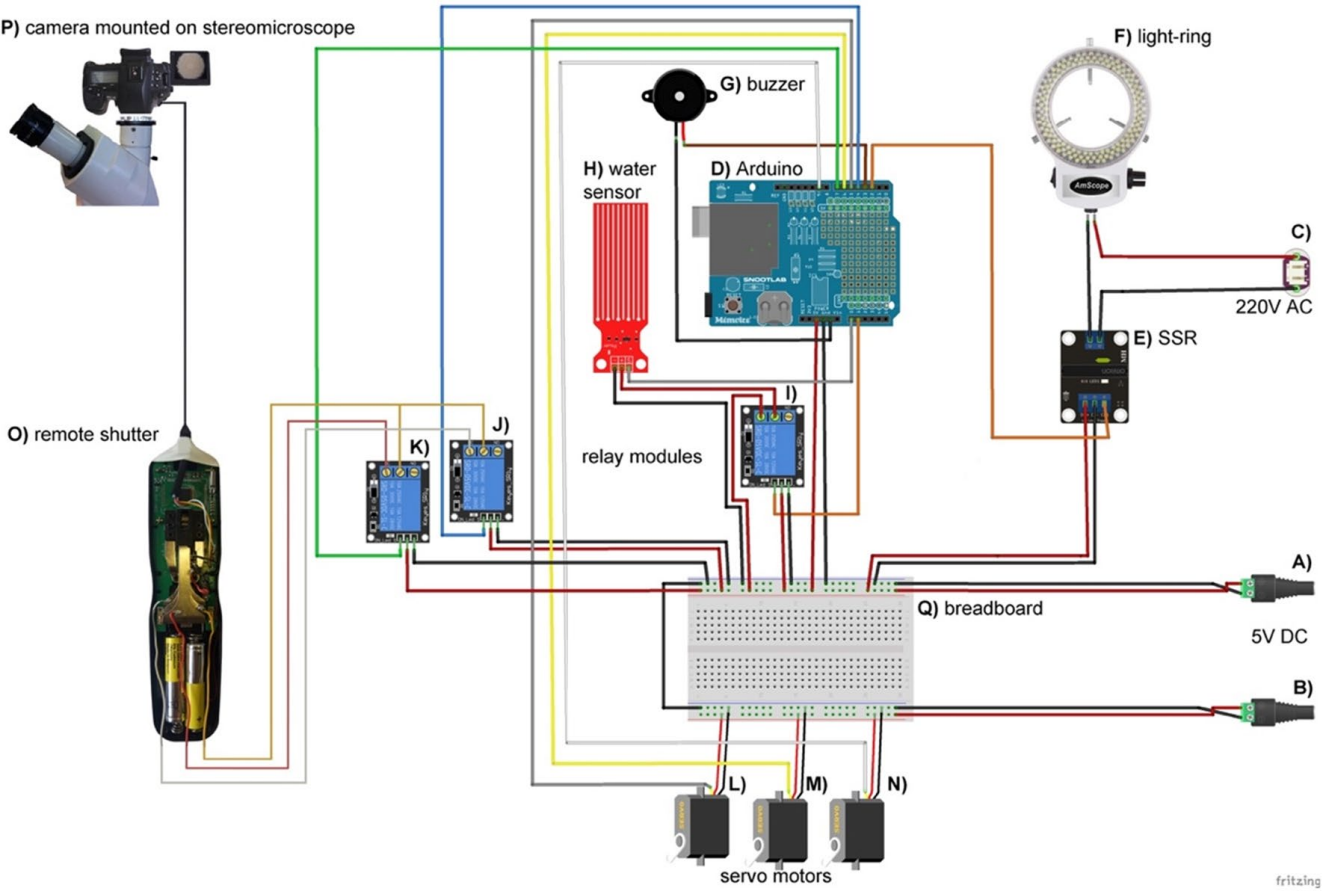
Arduino microcontroller wiring schematics. (**A,B**) 5 V DC power sources, (**C**) 20 V AC power source, (**D**) Arduino Uno R3 microcontroller board, (**E**) solid state relay module (Omron), (**F**) 144 LED light-ring for stereo microscopes (AmScope), (**G**) passive buzzer, (**H**) water level detection sensor module, (**I**–**K**) 5 V DC Arduino KY-019 relay modules, (**L**–**N**) SG90 servo motors, (**O**) digital timer and remote shutter MC-36B (Neewer), (**P**) SONY alpha 7 II mirrorless camera mounted on a Zeiss Stemi C-2000 stereo microscope using a T2 adapter, (**Q**) 30-row solderless breadboard with two bus stripes. Arduino schematic was developed using the Fritzing software V0.9.3^27^ and Adobe Photoshop CC 2017.

During the whole experiment the robotic arm is firmly attached to a Petri dish lid. In the resting periods—i.e., before the start of the experiment, after the last photo has been taken, and between the two recording steps—the robotic arm keeps the lid in a closed Petri dish position (Fig. 3B, Supplementary Video 2), thus preventing unnecessary medium desiccation and contamination. The Arduino microcontroller starts the time-lapse recording sequence by switching on the LED light-ring mounted on the objective of the stereomicroscope (Figs. 2E, 4F, Table 1, Supplementary Video 2) via the solid-state relay module (Fig. 4E).

**Table 1:** Approximate timeline intervals of a time-lapse recording cycle.

Steps of a recording cycle	Duration
Turning the LED ring ON	1 s
Raising the robotic arm/opening the Petri dish	2 s
Rotating the robotic arm out of the image acquisition path	2 s
Image acquisition	3 s
Rotating the robotic arm back	2 s
Lowering the robotic arm/closing the Petri dish	2 s
Turning the LED ring OFF	1 s
Checking the water-level and sounding the alarm if needed	1 s
Total active time	14 s
Waiting time (adjustable in the code)	5 min 46 s
Total recording cycle time	6 min

One second later, the robotic arm attached to the lid first moves several centimeters vertically, and then performs a side move to get the lid out of the camera’s field of view (Fig. 3C, Table 1, Supplementary Video 2). During these movements the lid keeps an approximately parallel position to the Petri dish that contains a growing biofilm. At the moment when the robotic arm stops in the opened Petri dish position (Fig. 3C, Table 1, Supplementary Video 2), the microcontroller takes a photo of the biofilm via the remote shutter (Fig. 2J, 4O) connected to the camera (Fig. 2E, 4P). After the photo has been recorded, the Petri dish is closed again by the same robotic arm movements, but in the opposite direction. One second after the closed Petri dish position is reached, the Arduino microcontroller switches off the light-ring (Table 1, Supplementary Video 2). The total time required for one arm movement cycle is around 14 s, a value that we empirically adjusted (Table 1). In our experience, faster arm movements lead to lower motion accuracy and consequently dysfunctional behavior. The next cycle of time-lapse recording is repeated depending on the time interval entered in the code that drives the microcontroller (Table 1).

To further minimize the dehydration of agar media during the filming of biofilm growth, we placed four square-shaped plastic containers filled with distilled water on the bottom of the incubator covering approximately 78% of the bottom area. These water-filled containers were performing the role of a humidifier, which allowed us to maintain relative air humidity within the incubator at approximately 85% during the visualization experiments that lasted up to 21 days. In the case a longer experimental growth period would be needed, our setup can be easily upgraded with the previously described double-decker agar hydration design^23^. Although relatively high humidity could potentially compromise functions of the electronic components in our setup, we had no problems of this type during the two years of its development and application. Nevertheless, the further improvements of the apparatus could include a protective enclosing for the microcontroller and the camera to prevent potential humidity-related issues.

To control the water level in water containers, we connected a water-level sensor to the Arduino microcontroller (Figs. 2K, 4H). In situations when the water level dropped below the critical threshold and the water containers needed refilling, the Arduino microcontroller sounded an alarm over the passive buzzer (Fig. 4G). We prevented unnecessary electrode corrosion of the water-level sensor through electrolysis by connecting it through a relay module (Fig. 4I), which switched on the sensor only during the measurement time.

**Figure 5:**
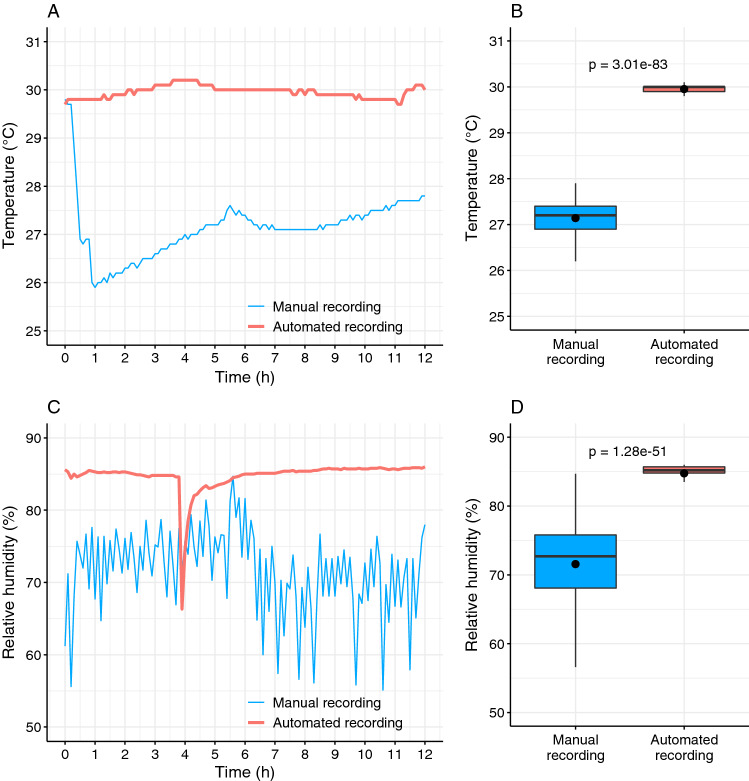
Temperature and humidity fluctuations during manual and automated time-lapse photo recording. To show the advantage of our automated system over manual time-lapse recording, we added the temperature and humidity sensors to the setup (Supplementary Fig. 1) and recorded temperature (**A**) and humidity fluctuations (**C**) during 12 h of the automated and manual time-lapse filming process. The average temperature (**B**) and humidity (**D**) values are shown in box-plots (black dots). During the course of automated filming process temperature and humidity values were measured every 6 min. The incubator door was continuously closed except for one focus adjustment and water container refill at 3 h and 54 min. In contrast, during the manual recording process we opened the incubator door, manually removed the Petri dish lid, took a photo, closed the Petri dish, closed the incubator door and measured the temperature and humidity. This process, which lasted approximately 10 s, was repeated every 6 min. We collected in total 121 temperature and humidity measurements per experiment (Supplementary File 1). We tested difference in the average temperature using two-tailed t-test. Corresponding p-values are shown in panels (**B**) and (**D**).

In order to detect an occasional camera focus loss due to biofilm growth in three dimensions and to prevent eventual mechanical misbehaviors of the system, it is necessary to occasionally control the activity of the setup during the filming session. However, to avoid the frequent opening of the incubator door, which in turn leads to temperature, humidity and lighting fluctuations within the incubator chamber (Fig. 5), we connected an external monitor to the camera that allows real-time monitoring of biofilm growth and setup behavior (Fig. 2B). As our camera can simultaneously take shots and provide a live image on the external monitor over a HDMI socket, there was no need to manually switch between these two modes. The real-time image of the biofilm was visible on the monitor only during the recording cycle because the light is only turned on during this period, whereas during the resting period between the recording cycles the incubator chamber is in dark. Nevertheless, this periodic availability of the real-time image was sufficient for the eventual inspection of biofilm growth or apparatus malfunctions.

Before the start of our visualization experiments, we sterilized the inner parts of the incubator, its atmosphere and the whole apparatus by a UV sterilizer that we added in the incubator chamber (Fig. 2D). This procedure allowed us to largely prevent contamination of agar media during the experiments. Nevertheless, we had to occasionally open the incubator during the experiment to manually correct the focus of the stereomicroscope that became suboptimal due to changes in the biofilm 3D morphology, or to refill the water containers. In the vast majority of experiments this was not a problem, but occasionally a spore contamination was unavoidable. However, from our experience, the air-borne fungal contamination was not visible on the agar-plates before two weeks of continuous recording (Supplementary Video 3). In order to further minimize the fungal or bacterial spore contamination of the agar-plates and temperature and humidity fluctuations (Fig. 5), an automated stereomicroscope focusing mechanism, as well as an automated water container refill mechanism, should be added to the setup in the future.

### Proof of concept: Strain-specific development in Bacillus subtilis

**Figure 6:**
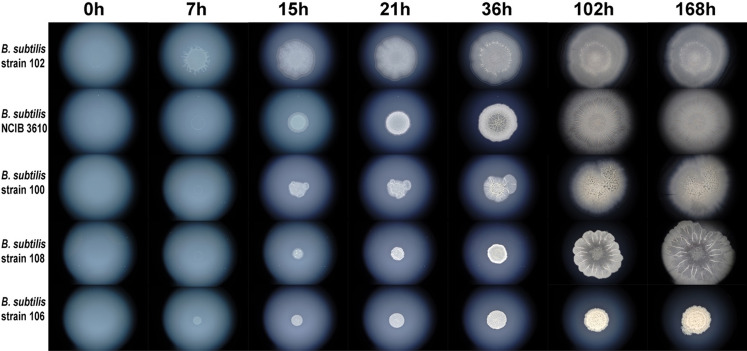
Selected time-lapse photos of developing *B. subtilis* biofilms. We recorded the biofilm development of five *B. subtilis* strains (102, NCIB3610, 100, 108, 106) using our time-lapse setup. Corresponding photos in seven time points are shown (0 h, 7 h, 15 h, 21 h, 36 h, 102 h, 168 h). The full time-lapse recordings for every strain that covers a seven day period are available in Supplementary Videos 3, 4, 5, 6 and 7. These time-lapse recordings reveal that five *B. subtilis* strains show a remarkable difference in developmental dynamics and biofilm morphology.

To demonstrate our time-lapse visualization method, we recorded developmental and morphological changes during the biofilm growth of five distinctive *B. subtilis* strains (Fig. 6).

We independently photographed the biofilm development of every strain in six-minute intervals. This recording frequency allowed us to produce detailed time-lapse videos with an optimal frame rate (30 fps). We recorded 1,681 photos for every of the four *B. subtilis* strains (102, 100, NCIB3610, and 108) that we grew for seven days. In addition, we collected 5,041 photos of a slowly developing strain (106) that we grew for 21 days. To give an overview of the recording process, we show seven representative photos recorded during seven-day period for all five strains (Fig. 6). However, as the full dynamics of biofilm growth and the changes in its morphology are only possible to grasp from time-lapse videos, we also assembled our photo collections into five time-lapse videos that show the biofilm growth of every strain independently (Supplementary Videos 3, 4, 5, 6 and 7). For comparative purposes, we also compiled two additional videos that show the growth of two strains (102, 106) and four strains (102, 100, NCIB3610 and 108) in parallel (Supplementary Videos 8 and 9). Finally, to show that our method has enough processivity to allow recording in replicates, which could be useful in assessing the morphological variability of biofilm development within a strain, we filmed the growth of strain 108 three times. We assembled these three time-lapse photo collections, which covered biofilm growth over three days, into three independent time-lapse recordings and joined them together into a single video (Supplementary Video 10).

The first obvious difference among the observed *B. subtilis* strains, which could be grasped by browsing their biofilm videos in a slow-motion mode, was their growth speed (Fig. 6, Supplementary Video 8). Although all observed strains grew under the same standardized environmental conditions (1.5% w/v agarose, 30 °C and 85% relative humidity), they showed rather different growth dynamics (Fig. 6, Supplementary Video 8 and 9). For instance, strain 102 showed the fastest biofilm expansion, in contrast to the 106 strain which showed the slowest growth within the seven-day timeframe (Fig. 6, Supplementary Video 9). Except the fastest growth, strain 102 showed a specific star-shaped morphology in the early phase (7 h) of biofilm growth that was absent in other strains (Fig. 6, Supplementary Videos 5, 8 and 9).

Besides the growth rate, in our time-lapse videos one could also look at the changes in colony morphology, which is considered one of the hallmarks of *B. subtilis* biofilms^28^. In our previous work we showed that typical wrinkled biofilm morphology in the NCIB3610 strain starts to develop in the transition stage during the second day of biofilm growth, which correlates with dramatic changes in expression patterns^14^. In this study we were able to reproduce this pattern in the same strain using our automated apparatus recording. The time-lapse video shows that wrinkles first start to appear in the center of the NCIB3610 biofilm at the onset of the second day of biofilm growth (Supplementary Video 7). In comparison to the NCIB3610 strain, the wrinkles in other four strains showed different morphology in terms of density, timing of their first appearance, and three-dimensional structure (Fig. 6, Supplementary Videos 8 and 9). This developmental variability between strains suggests differences in underlying expression patterns that could be further explored by time resolved sampling and subsequent transcriptomic and proteomic analysis^14^. In contrast to differences between the strains, the visual comparison of three replicates (strain 108) showed highly comparable morphological dynamics during the three-day biofilm growth (Supplementary Video 10).

### Advantages of automated over manual biofilm growth time-lapse recording

**Figure 7:**
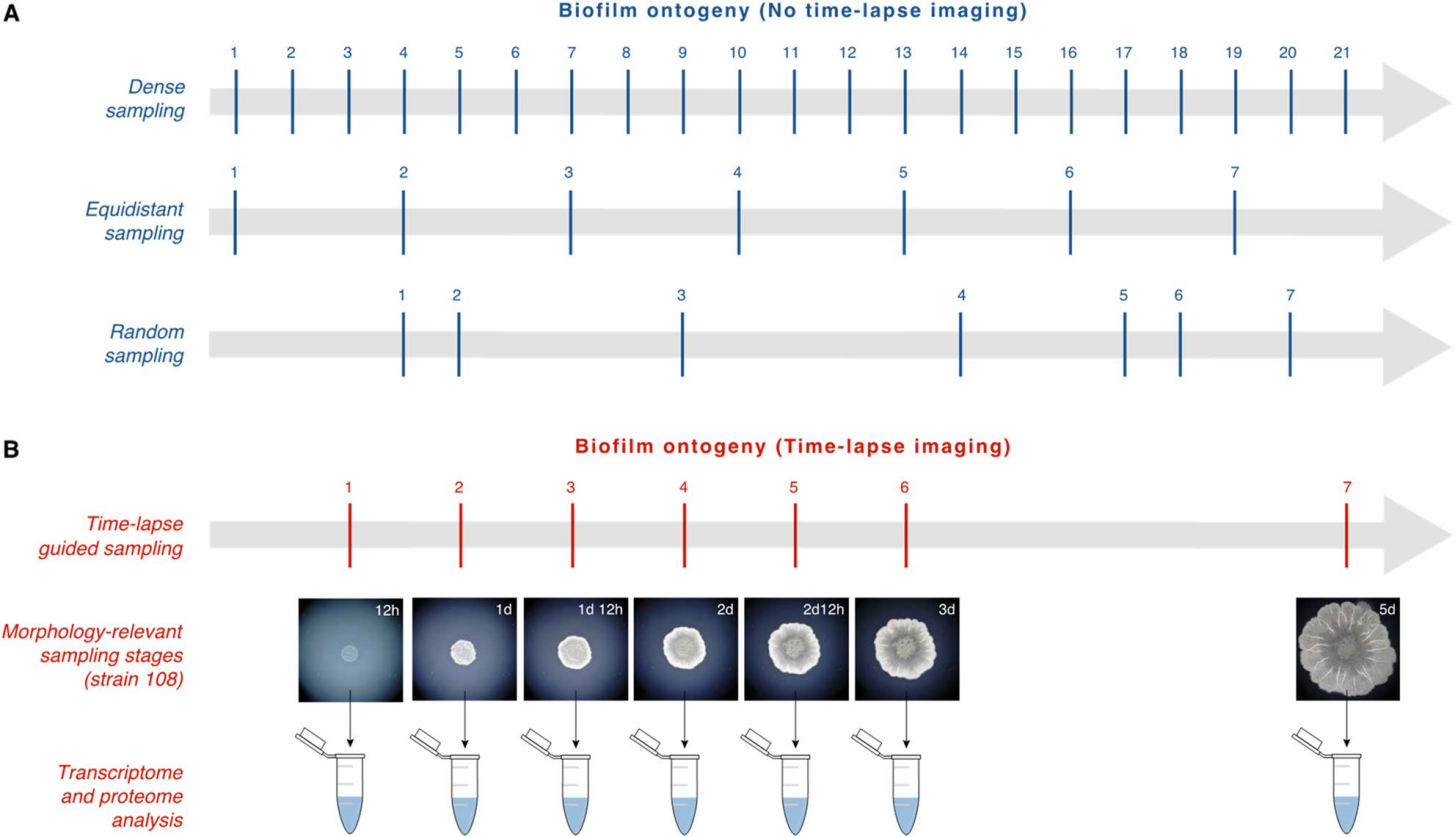
Different sampling strategies of biofilm development for downstream omics analysis. **(A)** We depicted some of the possible sampling strategies when a time-lapse recording of biofilm development is not available. Dense sampling is the best approach, but incurs high costs in downstream experiments, e.g. transcriptome sequencing and protein quantification, especially if the sampling includes replicates and covers long biofilm ontogenies. If the number of sampling timepoints is limited for any reason, *e.g.* to seven samples, one could choose an equidistant or eventually random sampling approach. In both cases, it is uncertain how these samples will reflect underlying expression dynamics. (**B**) In the case of limited number of samples, we suggest that a better option is to follow morphological change of biofilm development and to adjust sampling timepoints accordingly. By browsing time-lapse videos, timepoints relevant for biofilm development could be determined. For instance, we analyzed here the time-lapse video that covers biofilm development of the *B. subtilis* strain 108 during seven days (Supplementary Video 6). We selected seven time-points that we consider relevant for sampling: 12 h—early biofilm development, the inoculation droplet circle is visibly filled with cells, the first regions with ticker cell layers already start to appear; 1d—the cells start to spread outside of the inoculation droplet circle to from a ring, at the edge of inoculation droplet circle an irregular ridge made of ticker cell layers is formed; 1d 12 h—the ring continues to enlarge, the outer edge of the ring starts to thicken, inner circle shows additional ridges and wrinkles; 2d—the ring further continues to enlarge, first radial wrinkles across the ring start to appear, the outer belt of the ring becomes the thickest structure; 2d 12 h—the ring further continues to enlarge, radial wrinkles become prominent, inner part of the biofilm becomes obviously thinner compared to the outer belt; 3d—the biofilm continues to grow, has lace appearance, shows strong radial wrinkles and a curvy edge; 5d—at the upper-right sector of the biofilm edge a secondary growth becomes visible.

The main incentive for the development of our automated time-lapse imaging method was the simplification and improvement of biofilm sampling for downstream transcriptomic and proteomic experiments^14^. The choice of a sampling timeline in developmental expression studies is a sort of a multi-objective optimization problem where one looks to recover a maximal overall expression variance with a minimal number of samples. On the other hand, recovered expression variance depends on the temporal distribution of samples along biofilm ontogeny because biofilm development is a non-linear process^14^. This entails that it is very unlikely that optimal results would be achieved by simply taking a limited number of randomly or evenly distributed samples along biofilm ontogeny (Fig. 7A).

Hypothetically, one could make a very dense equidistant sampling to avoid the problem (Fig. 7A). However, the costs of transcriptomic and proteomic experiments additively grow with the number of samples, especially when replicates are included in an experimental design. Regardless of the costs, it is good to keep the total number of the samples at reasonable level because a large number of samples impose substantial computational burden on the bioinformatic pipelines for transcriptomic and proteomic analysis. However, without knowing in advance the global gene expression profiles, it is hard to optimally decide on the temporal distribution of biofilm sampling. Inspired by expression studies in animals^16^, we reasoned that if we follow morphological changes during biofilm growth, we will probably catch the most important transition in gene expression levels. Indeed, in Futo et al. we showed that morphology and expression profiles generally correlate during *B. subtilis* development^14^.

However, in this previous study, we faced the problem of filming biofilm gross morphology over long periods. As we already explained, water condensation on the lid was the major problem, but we also had difficulties to properly set the focus and lightning conditions through the lid. We ad hoc solved this problem by manually opening and closing the Petri dish during the filming of biofilm growth. This strategy worked, but at very high manpower cost, which prevented us from regularly applying it (Table 2). In addition, we struggled to keep temperature and humidity values during filming session as close as possible to the levels maintained during biofilm growth for omics sampling (Fig. 5). Our automated apparatus for time-lapse biofilm growth recording largely solved these problems. By reducing more than 30 times the manpower costs (Table 2), it makes time-lapse recording more processive and reproducible, while by stabilizing temperature and humidity values during the filming process (Fig. 5), it helps in keeping comparable environmental conditions between the biofilm sampling and filming experiments.

**Table 2:** Workload comparison between manual and automated biofilm time-lapse photo recording (7-day time-lapse movie).

Timeline	Procedure	Manual recording (man-hour)	Automated recording (man-hour)
1st day	Preparation of growth media (liquid and solid)	2	2
2nd day	Plating-out the frozen stock culture	0.25	0.25
3rd day	Preparation and inoculation of overnight culture	0.25	0.25
4th day	Plate inoculation and start of photo recording	24	0.1
5th day	Recording photographs with focus correction	24	0.1
6th day	Recording photographs with focus correction	24	0.1
7th day	Recording photographs with focus correction	24	0.1
8th day	Recording photographs with focus correction	24	0.1
9th day	Recording photographs with focus correction	24	0.1
10th day	Recording photographs with focus correction	24	0.1
11th day	Photo-processing (time tagging and scale-bar)	1	1
11th day	Assembling the photographs into a time-lapse video	1.5	1.5
	Total workload	173	5.7

### Time-lapse videos in guiding biofilm sampling for omics analysis

To detect relevant time points that show morphological change, in our previous work^14^ we visually browsed back-and-fort through a time-lapse movie of biofilm growth in a slow-motion mode. In this process we relied on the visual pattern recognition of a trained researcher because this is how morphological developmental changes were usually defined in animal development. As an example of this procedure, we selected the time points of biofilm development (strain 108) which we think are suitable for analysis in downstream transcriptomic and proteomic experiments (Fig. 7B).

However, we are also aware that bacterial biofilms are especially amenable for morphometric analysis, which could move the decision-making process related to biofilm sampling from a qualitative to quantitative level. In contemporary studies biofilm morphometrics is predominantly performed on 3D fluorescence images that are gathered by confocal microscopy^29^. Consequently, the state-of-the-art morphometric software, like BiofilmQ, is mainly designed for the analysis of these image types^29^. Although confocal microscopy and light sheet microscopy^30^ are powerful visualization techniques that are applied for biofilm analysis^25,29,31^, they require florescent labeling and a substantially more sophisticated setup. Nevertheless, from the perspective of our application, which aims to improve biofilm sampling, these prerequisites may represent a disproportionate effort. In addition, transgenic florescent labeling is not always possible to perform, for instance in environmental bacterial strains where genetic protocols have not been established. In this context, it was necessary to first check if our 2D images obtained by bright field stereomicroscopy with episcopic illumination were suitable for processing in BiofilmQ software.

**Figure 8:**
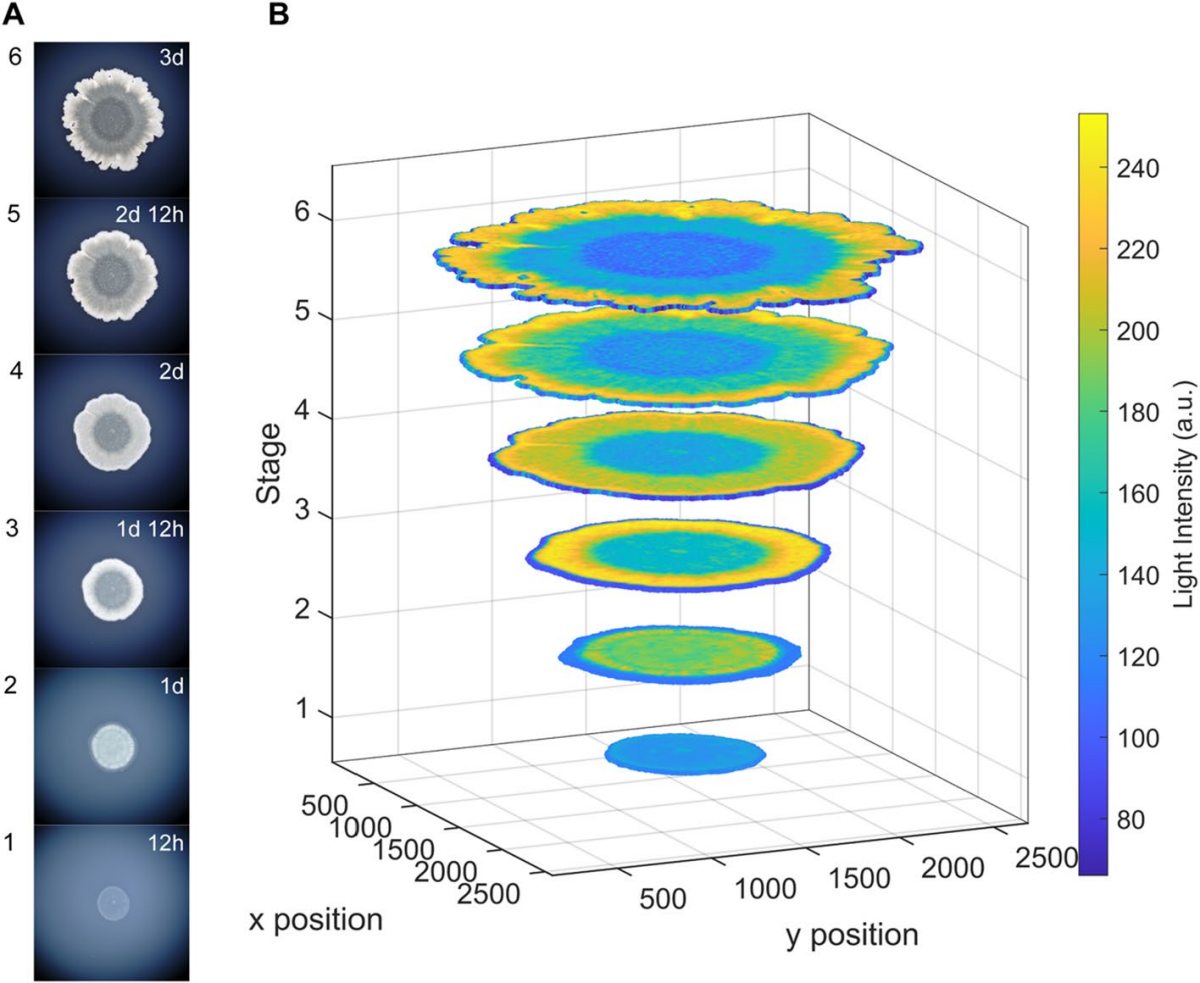
An example of quantitative biofilm image analysis. (**A**) Six images of developing *B. subtilis* biofilm (strain 108) gathered by bright field stereomicroscopy with episcopic illumination. We extracted these six images from three-day time-lapse video (replicate No. 2, Supplementary Video 10). The developmental timepoints of these images correspond to the timepoints that we marked as morphology-relevant for the strain 108 in Fig. 7B. (**B**) 4D scatterplot generated in BiofilmQ—a software for quantitative biofilm analysis and visualization^29^. We first pre-processed and segmented six biofilm images in BiofilmQ (see Methods) and then depicted the spatial position of the resulting squares (pseudo-cells) and their corresponding light intensity values (arbitrary unit). Under assumption that square-specific light intensity reflects biofilm thickness at a particular spatial position, it is clear that after one day of biofilm development the center of the biofilm is increasingly getting thinner (deep blue color), while a belt shaped region located close to the outer biofilm edge becomes the thickest part (bright yellow color).

To test this, we took six representative images obtained during time-lapse filming of the 108 strain (replicate No. 2, Supplementary Video 10, Fig. 8A), imported them into BiofilmQ and performed segmentation of their biosurface. Segmentation is a critical process in the image analysis of biofilms that partitions an image into a biologically relevant part (biofilm) and the rest of the image (background and noise)^29,32^. An overly pronounced background, low image sharpness, unintended objects and any kind of noise are factors that all decrease and complicate the segmentation process. However, it turned out that the segmentation of our time-lapse biofilm images, due to their visual quality, is quite straightforward. As an output the segmentation process in BiofilmQ yields a collection of squares (pseudo-cells) that define the biological object (biofilm). These squares have quantitative attributes like spatial position and light intensity that could be further processed. As an example, by using BiofilmQ visualization module we made a 4D scatterplot that shows the spatial position of squares (pseudo-cells) and their corresponding light intensity values for six selected stages of biofilm growth (Fig. 8B). Under the assumption that a square-specific light intensity value reflects the biofilm thickness at a particular spatial position, it is clear that after one day of biofilm development the center of the biofilm is increasingly getting thinner (deep blue color). Altogether, this demonstrates that images obtained with our automated time-lapse recording apparatus for bright field stereomicroscopy with episcopic illumination are suitable for downstream morphometric analysis. Moreover, the increased processivity of our automated time-lapse filming method, compared to the manual approach, opens possibility for a more detailed and robust morphometric analysis.

## Conclusions

We developed a novel automated time-lapse imaging method for studying the gross morphology of developing bacterial biofilms over long time periods, which solves the problem of condensation-induced photo blurriness in bright filed stereomicroscopy with episcopic illumination. As our method is based on an easily programmable Arduino microcontroller platform, it is highly flexible in terms of controlling photo periods for time-lapse photography. In addition, our setup could be easily upgraded with various additional elements such as diverse kinds of sensors that might come useful in different experimental setups. Although our visualization setup is primarily developed for visualization of biofilms or macrocolonies that grow on solid-air interfaces, with minimal modifications it could be also used for visualization of biofilms that float on liquid–air interfaces as pellicles. Due to the relatively low cost of its main components, this method is an affordable solution for microbiological time-lapse recording in various scientific and educational environments. The quality of the time-lapse videos that could be produced by this setup was already acknowledged with an Honorable mention award at the 2021 Nikon Small World in Motion Competition^33^.

## Material and methods

### Bacterial strains

#### Isolation, identification and storage

For the purpose of time-lapse visualization, we used five strains of the bacterial model species *Bacillus subtilis.* A widely-used, biofilm-forming *B. subtilis* subsp. *subtilis* str. NCIB3610 (strain 3610) was obtained from the Bacillus Genetic Stock Center (BGSC, Ohio State University, Columbus, OH, USA) and stored in 25% glycerol stocks at − 80 °C. Four additional *B. subtilis* environmental strains were isolated from a topsoil sample collected from Ruđer Bošković Institute backyard following the procedure described below. The five grams of topsoil were mixed with 45 mL of re-distilled, filter-sterilized water and vortexed for 3 min in a 50 mL falcon tube at room temperature (RT). After the soil precipitate has sedimented for 10 min at RT, the supernatant was serially diluted in 10^–1^, 10^–2^ and 10^–3^ steps. 100 μL of the undiluted and three diluted supernatants were plated out on 90 mm Petri dishes containing biofilm-promoting MSgg agar (5 mM potassium phosphate pH 7, 100 mM MOPS pH 7, 2 mM MgCl_2_, 700 μM CaCl_2_, 50 μM MnCl_2_, 50 μM FeCl_3_, 1 μM ZnCl_2_, 2 μM thiamine, 0.5% glycerol, 0.5% glutamate, 50 μg/mL tryptophan, 50 μg/mL phenylalanine solidified with 1.5% agar) using a Drigalski spatula in 10 replicates each. In total 40 inoculated MSgg plates were incubated for 24 h at 30 °C. After incubation, all plates were visually inspected using a Stemi C-2000 stereo microscope (Zeiss). In total, 14 single-standing colonies that showed a morphologically elaborate colony structure were isolated and inoculated on a fresh MSgg plates, each in three replicates. After 24 h of incubation at 30 °C, a single colony from each of the 14 samples was taxonomically analyzed using the MALDI-TOF Biotyper mass spectrometry system (Bruker Daltonik). To perform taxonomic identification this instrument compares the mass spectrum of a focal microorganism with a reference library, and returns spectral similarity scores. These spectral similarity scores were calculated using the Bruker's pattern-matching algorithm^34^. According to the manufacturer, a similarity score of ≥ 1.80 represents a high confidence match. In practice, numerous studies have shown that score values ≥ 2.0 provide accurate identification at the species level^35,36^. We annotated four samples that had top-two matches to *B. subtilis* with score values above 2.0 as *B. subtilis* and used them for the downstream visualization experiments. These isolated strains were arbitrary named as strain 100, 102, 106, and 108 and were stored in 25% glycerol stocks at -80 °C.

#### Growth conditions before and during the visualization experiments

Two days before each visualization experiment, the bacteria from the − 80 °C glycerol stock were plated on a LB agar plate (1% Bacto tryptone, 0.5% Bacto yeast extract, 1% NaCl, 1 mM NaOH solidified with 1.5% agar) and incubated for 24 h at 30 °C. 10 mL of liquid LB medium were inoculated with a single colony and incubated overnight (16 h) in a shaker at 30 °C and 250 rpm. On the day of the experiment, the plastic bottom of a 90 mm Petri dish containing MSgg agar was evenly painted with black acrylic paint (Marbau GmbH & Co. KG) and left upside down on the bench to dry on RT. After the paint had dried, the Petri dish was placed in the incubator, centered under the stereomicroscope objective (Zeiss Stemi C-2000, Figs. 2E, 3A) and glued to the stage plate of stereomicroscope with three drops of silicone glue. The incubator was closed and sterilized with a 254 nm OFR UV light for 30 min (Fig. 2D). Following the incubator surface-sterilization, the Petri dish lid was removed and the MSgg agar plate was inoculated with 5 μL of the bacterial overnight culture. With the help of live image projected on the external monitor (Fig. 2B), we pipetted this 5 μL drop in the middle of the field of view and focused the image using the focus nob of the stereomicroscope. Subsequently, the inoculated plate was covered with a Petri dish lid attached to the robotic arm (Fig. 3B, Supplementary Video 2). We started the experiment by turning the Arduino microcontroller ON and closing the incubator. During the filming experiments, biofilms were grown at 30 °C, for 3 days (strain 108 in three replicates), 7 days (strains 100, 102, 108 and NCIB 3610), and 21 days (strain 106), while the relative air humidity inside the incubator was maintained at around 85%.

### Arduino controlled time-lapse photography setup

#### The robotic arm

We performed visualization of five *B. subtilis* strains using the time-lapse automated setup (Figs. 2 and 4). The whole setup was operated by an Arduino Uno R3 microcontroller board (Arduino) (Fig. 4D). The Arduino digital pins 5, 6 and 9 were connected to three SG90 servomotors (Fig. 4L–N). The servomotors were moving a simple, acrylic robotic arm in three axes. We purchased this robotic arm from Keyestudio (https://www.keyestudio.com/products/keyestudio-4dof-acrylic-toys-robot-mechanical-arm-claw-kit-for-arduino-diy-robot) and assembled it according to the provided instructions (https://wiki.keyestudio.com/Ks0198_keyestudio_4DOF_Robot_Mechanical_Arm_Kit_for_Arduino_DIY). To allow a direct attachment of the claw servo plate to the lid of Petri dish with an adhesive tape we did not attach the apical robotic claw and its servomotor. All three servomotors were powered by a DC 5 V power-source (Fig. 4B) running separately from the main DC 5 V line that powered the microcontroller and the modules (Fig. 4A).

#### Light, temperature and humidity

To achieve optimal lightning conditions, we mounted an episcopic 144 LED light-ring (Fig. 4F) on the stereomicroscope objective (Figs. 2E, 3). The microcontroller-mediated solid-state relay module (Omron) (Fig. 4E), which was opening and closing the DC 12 V light-ring circuit (Fig. 4C), was controlling the light-ring ON/OFF switching. This relay was connected to the microcontroller via digital pin 2.

The optimal temperature for bacterial growth was provided by the microbiological incubator (Termomedicinski aparati—TMA) (Fig. 2A). All bacterial strains were grown at 30 °C. The air-humidity in the incubator was maintained by evaporation from four water containers placed in the incubator and filled with redistilled water (Fig. 2K). The water level in the containers was controlled with a water level sensor (Fig. 4H). The sensor was connected to the microcontroller’s analog input pin A0. The 5 V DC current powering the sensor was periodically switched ON/OFF by the 5 V DC KY-019 relay module (Fig. 4I). The relay module was connected to the analog input pin A1 of the microcontroller. Digital pin 3 was connected to a passive buzzer (Fig. 4G). The humidity was monitored with a HM16 thermo/hygrometer (Bauer GmbH) placed within the incubator.

For the purpose of comparing temperature and humidity fluctuations within the incubator interior during manual and automated time-lapse photo recording (Fig. 5), we upgraded our Arduino system described in Fig. 4 with two additional elements (Supplementary Fig. 1). An Arduino-compatible data logging shield (Velleman), which enabled temperature and humidity measurements to be stored onto a SD card, was mounted onto the microcontroller board (Supplementary Fig. 1D), and a digital temperature and humidity sensor AM2302 DHT22 was connected via the logging shield to the Arduino digital pin 8.

#### The photography and camera settings

The photographs of growing biofilms were recorded using a SONY alpha 7 II mirrorless camera (Fig. 4P) mounted on a Stemi C-2000 (Zeiss) stereo microscope using a T2 adapter (Fig. 2E). Digital pins 4 and 7 of the microcontroller board were connected to two DC 5 V KY-019 relay modules (Fig. 4J,K). Once triggered by a signal received from the microcontroller, the two relay modules (Fig. 4K,L), one after the other with 1 s delay, closed the two 1.5 V DC circuits of the remote shutter MC-36B (Neewer) (Figs. 2J, 4O). This action imitated a physical finger-push on the shutter release trigger and as a result sent a signal to the camera via a USB connection. The photographs were taken in six-minute intervals during three days (strain 108 in three replicates), four days (strain 108 recorded through the Petri dish lid), seven days (strains 100, 102, 108 and NCIB3610) and 21 days (strain 106). The magnification of the stereomicroscope was set to 0.8 x. The camera was set to the “Aperture Priority” mode with the following parameters: image size: 24 M; image quality: RAW + J; image resolution: 6000 × 4000 px, 350 dpi; drive mode: single shooting; flash compensation: ± 0.0; focus area: center; exposure compensation: ± 0.0; light sensitivity ISO: 50; metering mode: multi; white balance: AWB; creative style—“Clear” while the D-Range Optimizer and flash were set to “off”.

### Production of time-lapse videos

The time-lapse videos of developing biofilm macrocolonies were produced from series of JPEG photographs using the Adobe After Effects CC 2017 software. The time-lapse videos of *B. subtilis* developing biofilms that correspond to 102, 100, NCIB3610 and 108 strains were rendered from four photo collections each containing 1,681 photographs. The time-lapse video of the 106 strain was rendered using 5,041 photographs, while the videos of the 108 strain that were recorded in three replicates were rendered from 721 photographs each. The rendering settings were set to: “Best”, video size was 1920 × 1080 px in full resolution, auto input was switched off. The frame rate in all videos was set to 30 fps, the video format was QuickTime, and the codec was MPEG-4.

### Quantitative analysis of biofilm images

The six TIFF images of the developing *B. subtilis* biofilm strain 108 (replicate No. 2, Supplementary Video 10) were imported into BiofilmQ^29^. We first performed image alignment (mean squares registration), then cropping, thresholding, and finally segmentation using "cubes" dissection method. The segmented 2D biosurface contained 4,690 (12 h), 10,122 (1d), 16,286 (1d 12 h), 24,179 (2d), 31,974 (2d 12 h) and 38,848 (3d) squares (pseudo-cells). Using single object parameters (centroid coordinate x, centroid coordinate y, image ID, and light intensity), for every square (pseudo-cell) we plotted 4D scatterplot in Fig. 8B.

## Supplementary Information

Supplementary Video 1.

Supplementary Video 2.

Supplementary Video 3.

Supplementary Video 4.

Supplementary Video 5.

Supplementary Video 6.

Supplementary Video 7.

Supplementary Video 8.

Supplementary Video 9.

Supplementary Video 10.

Supplementary Information 1.

Supplementary Information 2.

Supplementary Legends.

## Data Availability

The videos presented in this paper are available for immediate viewing on the private YouTube links and for viewing and download in the original size on the FigShare links provided in below table. The code driving the Arduino microcontroller and the Python script for photography time-tagging are available at the GitHub link https://github.com/bacillus-biofilms/time-lapse-imaging.

**Table:** 

Video name	Video shows	YouTube link	FigShare link
Supplementary Video 1	Strain 108/with lid	https://youtu.be/viO8-M122UA	https://figshare.com/s/430b9c7f34b0cd16b93a
Supplementary Video 2	Robot movements	https://youtu.be/Qc9HmVKegRg	https://figshare.com/s/6cd7e6823025d4710567
Supplementary Video 3	Strain 106	https://youtu.be/MLW_NvBZwPs	https://figshare.com/s/8d89f43bec93c11eeb0f
Supplementary Video 4	Strain 100	https://youtu.be/av_25ZMm_1s	https://figshare.com/s/9a5cd2cffeca5d204077
Supplementary Video 5	Strain 102	https://youtu.be/OJcraAnx76Y	https://figshare.com/s/fe26f8d2b5eb6edf0056
Supplementary Video 6	Strain 108	https://youtu.be/GGEYQy4pvHI	https://figshare.com/s/bcf52a5cc90cd89083a4
Supplementary Video 7	Strain NCIB3610	https://youtu.be/C9CgCAxGFIo	https://figshare.com/s/8dc5da67abc8f61c458f
Supplementary Video 8	Four strains	https://youtu.be/44c4pQeaWQM	https://figshare.com/s/6337518da2115684388f
Supplementary Video 9	Two strains	https://youtu.be/g6svu7HWZzk	https://figshare.com/s/9f6932242d9af894a8df
Supplementary Video 10	Strain 108/3 rep	https://youtu.be/Bmb-sTf24Vg	https://figshare.com/s/f8b806f3322bdce42f5c

## References

[1] Flemming H-C, Wuertz S (2019). Bacteria and archaea on Earth and their abundance in biofilms. Nat. Rev. Microbiol..

[2] Flemming H-C (2022). The biofilm matrix: Multitasking in a shared space. Nat. Rev. Microbiol..

[3] Flemming H-C, Wingender J (2010). The biofilm matrix. Nat. Rev. Microbiol..

[4] Arnaouteli S, Bamford NC, Stanley-Wall NR, Kovács ÁT (2021). Bacillus subtilis biofilm formation and social interactions. Nat. Rev. Microbiol..

[5] Yang L (2011). Current understanding of multi-species biofilms. Int. J. Oral Sci..

[6] Escudero C, Vera M, Oggerin M, Amils R (2018). Active microbial biofilms in deep poor porous continental subsurface rocks. Sci. Rep..

[7] Sauer K (2022). The biofilm life cycle: Expanding the conceptual model of biofilm formation. Nat. Rev. Microbiol..

[8] Burmølle M (2010). Biofilms in chronic infections: A matter of opportunity—monospecies biofilms in multispecies infections. FEMS Immunol. Med. Microbiol..

[9] Percival SL, Suleman L, Vuotto C, Donelli G (2015). Healthcare-associated infections, medical devices and biofilms: Risk, tolerance and control. J. Med. Microbiol..

[10] Backer R (2018). Plant growth-promoting rhizobacteria: Context, mechanisms of action, and roadmap to commercialization of biostimulants for sustainable agriculture. Front. Plant Sci..

[11] Cámara M (2022). Economic significance of biofilms: A multidisciplinary and cross-sectoral challenge. Npj Biofilms Microbiomes.

[12] Franklin MJ, Chang C, Akiyama T, Bothner B (2015). New technologies for studying biofilms. Microbiol. Spectr..

[13] Azeredo J (2017). Critical review on biofilm methods. Crit. Rev. Microbiol..

[14] Futo M (2021). Embryo-like features in developing *Bacillus subtilis* Biofilms. Mol. Biol. Evol..

[15] Domazet-Lošo T, Brajković J, Tautz D (2007). A phylostratigraphy approach to uncover the genomic history of major adaptations in metazoan lineages. Trends Genet..

[16] Domazet-Lošo T, Tautz D (2010). A phylogenetically based transcriptome age index mirrors ontogenetic divergence patterns. Nature.

[17] Shi L (2020). Evolutionary analysis of the *Bacillus subtilis* genome reveals new genes involved in sporulation. Mol. Biol. Evol..

[18] Campos-Ortega JA, Hartenstein V (1985). The Embryonic Development of Drosophila melanogaster.

[19] Kimmel CB, Ballard WW, Kimmel SR, Ullmann B, Schilling TF (1995). Stages of embryonic development of the zebrafish. Dev. Dyn..

[20] Monds RD, O’Toole GA (2009). The developmental model of microbial biofilms: Ten years of a paradigm up for review. Trends Microbiol..

[21] Levin M, Hashimshony T, Wagner F, Yanai I (2012). Developmental milestones punctuate gene expression in the Caenorhabditis embryo. Dev. Cell.

[22] Yanai I (2018). Development and evolution through the lens of global gene regulation. Trends Genet..

[23] PeñilCobo M (2018). Visualizing bacterial colony morphologies using time-lapse imaging chamber MOCHA. J. Bacteriol..

[24] Finer JE, Finer JJ (2007). A simple method for reducing moisture condensation on Petri dish lids. Plant Cell Tissue Organ Cult..

[25] Srinivasan S (2018). Matrix production and sporulation in bacillus subtilis biofilms localize to propagating wave fronts. Biophys. J..

[26] Nester, E. W. *Microbiology: A Human Perspective*. (McGraw-Hill, 2001). ISBN-13: 978-0072318784

[27] Knörig, A., Wettach, R. & Cohen, J. Fritzing: A tool for advancing electronic prototyping for designers. In *Proceedings of the 3rd International Conference on Tangible and Embedded Interaction—TEI ’09* 351 (ACM Press, 2009). 10.1145/1517664.1517735

[28] Branda SS, González-Pastor JE, Ben-Yehuda S, Losick R, Kolter R (2001). Fruiting body formation by *Bacillus subtilis*. Proc. Natl. Acad. Sci. USA.

[29] Hartmann R (2021). Quantitative image analysis of microbial communities with BiofilmQ. Nat. Microbiol..

[30] Stelzer EHK (2021). Light sheet fluorescence microscopy. Nat. Rev. Methods Primer.

[31] Qin B (2020). Cell position fates and collective fountain flow in bacterial biofilms revealed by light-sheet microscopy. Science.

[32] Berg S (2019). ilastik: Interactive machine learning for (bio)image analysis. Nat. Methods.

[33] Futo, M. *‘5-day time-lapse of Bacillus subtilis biofilm growth and development’ Honorable Mention Award at the 2021 Nikon Small World in Motion Competition*. https://www.nikonsmallworld.com/galleries/2021-small-world-in-motion-competition/time-lapse-of-bacillus-subtilis-biofilm-growth-and-development. (2021).

[34] Sauer S (2008). Classification and identification of bacteria by mass spectrometry and computational analysis. PLoS ONE.

[35] Rodriguez-Temporal D, Rodríguez-Sánchez B, Alcaide F (2020). Evaluation of MALDI biotyper interpretation criteria for accurate identification of nontuberculous mycobacteria. J. Clin. Microbiol..

[36] Tsuchida S, Nakayama T (2022). MALDI-based mass spectrometry in clinical testing: Focus on bacterial identification. Appl. Sci..

