# Supplementary material for: A novel time-lapse imaging method for studying developing bacterial biofilms
Source: Sci Rep. 2022 Dec 7;12:21120. doi: 10.1038/s41598-022-24431-y (PMC9729682; doi:10.1038/s41598-022-24431-y)
Supplement: Supplementary file 11 — Supplementary Information 1. [file 41598_2022_24431_MOESM11_ESM.docx]

**A novel time-lapse imaging method for studying developing bacterial biofilms**

Momir Futo, Tin Široki, Sara Koska, Nina Čorak, Anja Tušar, Mirjana Domazet-Lošo, Tomislav Domazet-Lošo^2,3^

***Supplementary material***


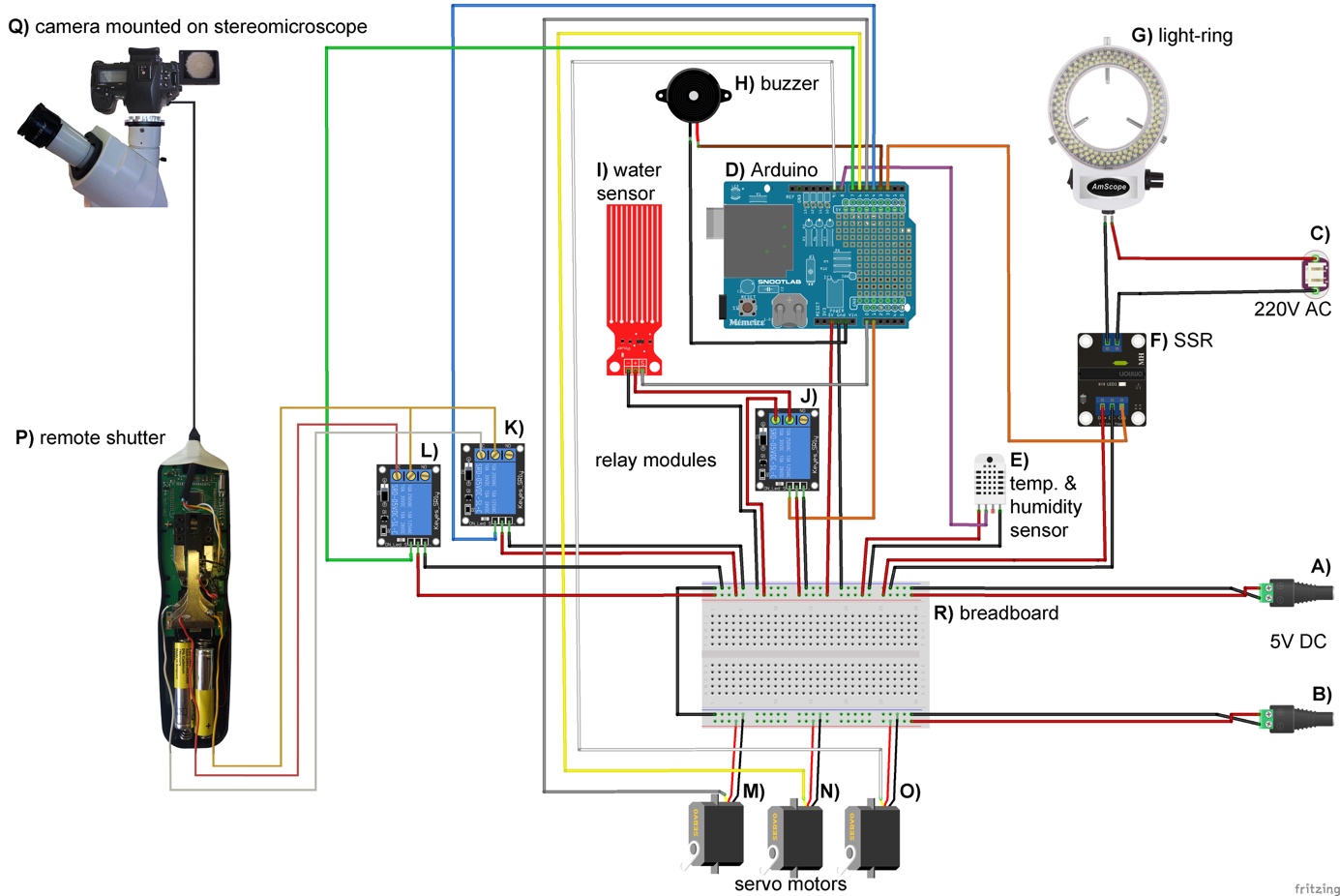


**Supplementary Figure 1. Arduino microcontroller wiring schematics with temperature sensor.** | **A, B** - 5V DC power sources, **C** – 220V AC power source, **D –** Arduino-compatible data logging shield (Velleman) mounted onto a Arduino Uno R3 microcontroller board, **E** – AM2302 DHT22 digital temperature and relative humidity sensor module, **F** – solid state relay module (Omron), **G** – 144 LED light-ring for stereo microscopes (AmScope), **H** - passive buzzer, **I** – water level detection sensor module, **J, K, L** – 5V DC Arduino KY-019 relay modules, **M, N, O** – SG90 servo motors, **P** – digital timer remote shutter release trigger MC-36B (Neewer), **Q** – SONY alpha 7 II mirrorless camera mounted on a Zeiss Stemi C-2000 stereo microscope using a T2 adapter, **R** – 30-row solderless breadboard with two bus stripes. Arduino schematic was developed using the Fritzing software V0.9.3^20^ and Adobe Photoshop CC 2017.
