# Supplementary material for: A novel time-lapse imaging method for studying developing bacterial biofilms
Source: Sci Rep. 2022 Dec 7;12:21120. doi: 10.1038/s41598-022-24431-y (PMC9729682; doi:10.1038/s41598-022-24431-y)
Supplement: Supplementary file 13 — Supplementary Legends. [file 41598_2022_24431_MOESM13_ESM.docx]

**Supplementary videos description**

**Supplementary Video 1. *Bacillus subtilis* biofilm growth recorded through a Petri dish lid.**

The time-lapse video of *Bacillus subtilis* biofilm growth during four days (strain 108). The time-lapse recording was performed through the Petri dish lid with observable condensation that obstructed the image quality. The video is available at https://youtu.be/viO8-M122UA and https://figshare.com/s/430b9c7f34b0cd16b93a.

**Supplementary Video 2. Robotic arm movements.**

Automated biofilm imaging system. The video demonstrates the movement of Arduino-controlled robotic arm during the time-lapse recording process. The video is available at https://youtu.be/Qc9HmVKegRg and https://figshare.com/s/6cd7e6823025d4710567.

**Supplementary Video 3. *Bacillus subtilis* biofilm growth (strain 106).**

The time-lapse video of the *Bacillus subtilis* biofilm growth during 21 days (strain 106). D: days, H: hours, M: minutes after inoculation. The scale bar indicates 1 cm. The video is available at https://youtu.be/MLW_NvBZwPs and https://figshare.com/s/8d89f43bec93c11eeb0f.

**Supplementary Video 4. *Bacillus subtilis* biofilm growth (strain 100).**

The time-lapse video of the *Bacillus subtilis* biofilm growth during seven days (strain 100). D: days, H: hours, M: minutes after inoculation. The scale bar indicates 1 cm. The video is available at https://youtu.be/av_25ZMm_1s and https://figshare.com/s/9a5cd2cffeca5d204077.

**Supplementary Video 5. *Bacillus subtilis* biofilm growth (strain 102).**

The time-lapse video of the *Bacillus subtilis* biofilm growth during seven days (strain 102). D: days, H: hours, M: minutes after inoculation. The scale bar indicates 1 cm. The video is available at https://youtu.be/OJcraAnx76Y and https://figshare.com/s/fe26f8d2b5eb6edf0056.

**Supplementary Video 6. *Bacillus subtilis* biofilm growth (strain 108).**

The time-lapse video of the *Bacillus subtilis* biofilm growth during seven days (strain 108). D: days, H: hours, M: minutes after inoculation. The scale bar indicates 1 cm. The video is available https://youtu.be/GGEYQy4pvHI and https://figshare.com/s/bcf52a5cc90cd89083a4.

**Supplementary Video 7. *Bacillus subtilis* biofilm growth (strain NCIB3610).**

The time-lapse video of the *Bacillus subtilis* biofilm growth during seven days (strain NCIB3610). D: days, H: hours, M: minutes after inoculation. The scale bar indicates 1 cm. The video is available at https://youtu.be/C9CgCAxGFIo and https://figshare.com/s/8dc5da67abc8f61c458f.

**Supplementary Video 8. *Bacillus subtilis* biofilm growth — comparative video of four strains.**

The comparative time-lapse video of biofilm growth during seven days. Four *Bacillus subtilis* strains (100, 102, 108 and NCIB3610) are shown. D: days, H: hours, M: minutes after inoculation. The scale bar indicates 1 cm. The video is available at https://youtu.be/44c4pQeaWQM and https://figshare.com/s/6337518da2115684388f.

**Supplementary Video 9. *Bacillus subtilis* biofilm — comparative video of two strains.**

The comparative time-lapse video of biofilm growth during seven days. Two *Bacillus subtilis* strains (102 and 106) are shown. D: days, H: hours, M: minutes after inoculation. The scale bar indicates 1 cm. The video is available at https://youtu.be/g6svu7HWZzk and https://figshare.com/s/9f6932242d9af894a8df.

**Supplementary Video 10. *Bacillus subtilis* strain 108 — three replicates.**

The comparative time-lapse video of biofilm growth during three days. The three replicates of *Bacillus subtilis* strain 108 are shown. D: days, H: hours, M: minutes after inoculation. The scale bar indicates 1 cm. The video is available https://youtu.be/Bmb-sTf24Vg and https://figshare.com/s/f8b806f3322bdce42f5c.
